# Solution proposal for completed preference structure in ORESTE method

**DOI:** 10.1038/s41598-023-31561-4

**Published:** 2023-03-23

**Authors:** Mehmet Akif Yerlikaya, Kürşat Yildiz, Büşra Nur Keskin

**Affiliations:** 1 Deparment of Industrial Engineering, Bitlis Eren University, Bitlis, Turkey; 2 Deparment of Civil Engineering, Gazi University, Ankara, Turkey; 3 Deparment of Traffic Planning and Application, Gazi University, Ankara, Turkey

**Keywords:** Scientific data, Statistics

## Abstract

In this study, a novel integrated method including CRITIC (Criteria Importance Through Inter-Criteria Correlation) and ORESTE (Organisation, Rangement Et Synth&e De DonnCes Relarionnelles) methods from MCDM (multi-criteria decision making) methods and aiming to solve the problem of inconsistency in the ORESTE method has been proposed. Since the ORESTE method only considers the ordering of alternatives and criteria, a conflict may occur due to the different ordering of each alternative in the criteria. To solve the conflict problem in the second step of the ORESTE method, it is proposed to create the preference density matrix of the alternatives by using the CRITIC weights of the criteria, and to calculate the net flow values with reference to the PROMETHEE II (Preference Ranking Organization Method for Encrichment Evaluations II) method (C-ORESTE III). The CRITIC method is used because the relationship between the criteria is as important as the alternatives in calculating the normalized preference intensities. To test the validity and applicability of the proposed method, an application is made to the logistics center location problem within the scope of green logistics.

## Introduction

Decision making is the act of choosing the correct pattern among alternative behavior patterns. A decision-making process includes the decision maker, alternatives, criteria, the decision maker’s priorities, objective priorities, environmental effects, and the consequences of the decision. The process may end with the decision maker choosing, ranking or classifying among the available options. According to this perspective, multi-criteria decision making (MCDM) methods are way of thinking which provides the right decision. Multi-criteria decision-making methods based on pairwise comparisons of certain criteria in general and it helps to make the most accurate decision with numerical data^1^. MCDM is the general name given to the solution of problems in which multiple and conflicting goals (criteria) are desired to be achieved. MCDM is seen as the fastest growing branch of Operations Research in recent years and represents an area that renews and revives the characters of systems thinking, multidisciplinary and scientific approach in problem solving, which is the essence of this field^2^.

In this study, a novel integrated method (C-ORESTE) including CRITIC and ORESTE methods from MCDM methods and aiming to solve the problem of inconsistency in the ORESTE method has been proposed. The CRITIC method is a method used to calculate the objective weights of the criteria considered in multi-criteria decision making problems, and it provides an important source of information to decision makers by obtaining important criterion weights called “Objective weights”. The ORESTE method is a ranking method in which the superiority of alternatives, also called “action”, is determined over another alternative according to each criterion^3^. This method simply ranks the alternatives without considering the value differences in each criterion set. Thus, it allows to compare alternatives on different sets of criteria. However, since this method only considers the ranking of alternatives and criteria, inconsistency may arise due to the different rank of each alternative in the criteria. In the second stage of the ORESTE method for the resolution of the conflict problem, it is suggested that the alternative density matrix is created by using the weights of the alternatives, and in the third step, the net flow values are calculated based on the PROMETHEE II method according to the preference density matrix (C-ORESTE). The preference density is the mean sequence of rank differences from one alternative to the other alternative based on each criterion. That is, it is the degree of preference according to the global order. The mean sequence is the sum of the order differences divided by the total number of transactions. The aim is to obtain more effective results by taking into account the degree of contradiction between criteria (CRITIC Weight) as the Global weak rank values are taken into account in determining the density of preference among the alternatives, and to complete the ranking of the alternatives by creating a new preference structure according to the net flow values. The preference structure indicates superiority, equality, or incomparability in pairwise comparison of alternatives. To test the validity and applicability of the C-ORESTE method, an application was made to the logistics center location problem within the scope of green logistics. Green logistics can be defined as a term that emerged intending to minimize the environmental problems that arise as a result of logistics activities^4^. This definition supports the importance of the concept of green logistics in practice to reduce environmental problems in the logistics sector and ensure sustainability. The data required for the application and the criteria taken into account in the selection of the green logistics location were obtained by conducting fieldwork with the help of an expert team within the framework of the concept of sustainability and environmentalism.

Studies using the ORESTE method reveal that the inability to use the net weight values of the criteria and the incomplete preference structure can be a problem for the decision-maker. Therefore, decision makers are looking for alternative methods or ORESTE hybrid methods. Therefore, the ORESTE method is not preferred much. The novelty of this study is that it both presents a completed preference structure and considers criterion weights in normalized transactions. Thus, it is expected that this method will be used more effectively.

The article is organized as follows: “Literature research” section provides studies on the ORESTE method; “Methodology” section highlights a proposed new integrated approach. Finally, the results are discussed in “Conclusions” section.

## Literature research

Although the ORESTE method is not widely used in the literature like the ELECTRE and PROMETHEE methods, it has been used in the solution of a limited number of decision problems. Since it is effective in the absence of numerical data on alternatives, it comes to the fore in decision-making problems. The ORESTE method was proposed by Roubens^5^ and later used by Roubens^6^ in a case study. Pastijn and Leysen^7^ also proposed a projection distance calculation method for the ORESTE method. Pastjin and Leener^8^ used the ORESTE and PROMETHEE method to select the best sensor combination for landmine detection strategies and compared the solution results. In addition to them, Givescu^9^ in the selection of the most suitable tourism areas to improve the country’s tourism performance, Matejček and Brožová^10^ in agricultural decision problems, Chatterjee and Chakraborty^11^ in the selection of materials, Jafari et al.^12^ port ranking, Eroǧlu et al.^13^ used the ORESTE method in personnel selection. Işık^14^ listed the insurance company alternatives using QUALIFLEX and ORESTE methods and compared the results. Yerlikaya and Arıkan^3^ handled the performance evaluation problem of supports with AHP, ORESTE and PROMETHEE methods so that SMEs (Small Medium Enterprises) can choose the support that will provide the best benefit to them, and they took into account the full ranking in PROMETHEE II for the preference structure that could not be completed in ORESTE II. Luo et al.^15^, probability-based hybrid ORESTE method was used to evaluate thermal comfort in underground mines. Wang et al.^16^ used the dual hierarchy hesitant fuzzy linguistic (DHLFS) ORESTE method to solve a problem involving city selection by evaluating the traffic density model. Zheng et al.^17^ evaluated failure mode and effects with the hybrid range type-2 fuzzy (ITTF) ORESTE method. Long and Liao^18^ made sustainable supplier selection using the q-step orthopyr fuzzy set distance ORESTE (q-ROF–SPAN–ORESTE) method. Akman et al.^19^ used Fuzzy AHP and ORESTE methods for supplier selection in the new product development process. Shi et al.^20^ proposed a new integrated approach by integrating trapezoidal fuzzy numbers (ITFN), combination weighting method and an extended ORESTE approach. They used the weak order for the missing preference structure in ORESTE II and evaluated the rock explosion risk of different lithologies in the Kaiyang phosphate mine. Zhang et al.^21^ developed an improved interval type-2 fuzzy ORESTE (IT2F-ORESTE) method based on distance and probability to assess regional economic correctability. Then, some comparative analyzes with other methods are made to show that the developed IT2F-ORESTE method can scientifically and accurately support the economic system recovery decision. Qin et al.^22^ proposed ORESTE-SORT in the classification of regional ports to determine the level of port group competitiveness.

In the literature, for the incomplete preference structure in the ORESTE method, some decisions were made according to the ORESTE II missing structure, while in some others ORESTE I and a different method were taken as reference. However, these studies considered the mean weight of the criteria in the ORESTE II normalizing procedure. In this study, a three-stage integrated method is proposed to solve the incomplete preference structure problem in the ORESTE method. For this, the CRITIC weights of the criteria were taken into account in the ORESTE II normalization process and net flow values were suggested for complete ranking. A literature summary comparing these studies with the proposed C-ORESTE is given in Table 1. The difference of this study from other studies is that it provides a complete preference structure by using CRITIC weights in the ORESTE method.

**Table 1:** Studies on the ORESTE method.

Author (year)	Methodology	Preference density	Incompleted preference structure	Completed preference	Application field
Pastijn and Leysen (1989)	ORESTE	Mean weighted (1/k)	ORESTE II	–	–
Pastjin and Leener (2002)	ORESTE and PROMETHEE	Mean weighted (1/k)	ORESTE II	–	Defense system
Givescu (2007)	ORESTE I	–	–	–	Tourism
Matějček and Brožová (2011)	ORESTE I	–	–	–	Agrıcultural decision problem
Chatterjee and Chakraborty (2012)	ORESTE I	–	–	–	Material selection
Eroğlu et al. (2014)	ORESTE I	–	–	–	Employee Selection
Işık (2016)	QUALIFLEX and ORESTE	Mean weighted (1/k)	ORESTE II	–	Insurance companies
Yerlikaya and Arıkan (2016)	AHP, PROMETHEE and ORESTE	Mean weighted (1/k)	ORESTE II	PROMETHEE II	SME supports
Luo et al. (2020)	Probabilistic ORESTE	Mean weighted (1/k)	ORESTE II	–	Undersoil ore and minerals
Wang et al. (2020)	DHLFS and ORESTE	Mean weighted (1/k)	ORESTE II	–	City selection
Zheng et al. (2021)	ITTF ORESTE	Mean weighted (1/k)	ORESTE II	–	Failure mode and effects analysis
Long and Liao (2021)	q-ROF–SPAN–ORESTE	Mean weighted (1/k)	ORESTE II	–	Supplier selection
Akman et al. (2021)	Fuzzy AHP and ORESTE	Mean weighted (1/k)	ORESTE II	–	Supplier selection
Shi et al. (2022)	TrFNs, entropy and ORESTE	Mean weighted (1/k)	ORESTE II	ORESTE I	Explosion risk assessment
Zhang et al. (2022)	IT2TF ORESTE	Mean weighted (1/k)	ORESTE II	–	The assessment of regional economic restorability
Qin et al. (2022)	ORESTE-SORT	Mean weighted (1/k)	ORESTE II	–	Sorting port group competitiveness
This study	C-ORESTE	CRITIC weighted (w_j_)	C-ORESTE II	C-ORESTE III	Green logistic location selection

## Methodology

### CRITIC and ORESTE method

#### CRITIC method

Diakoulaki et al.^23^ developed the CRITIC method based on Standard Deviation (SD), Average Weights (MW) and Correlation in order to weight the three evaluation criteria they used in their study where they measured the performance of eight Greek pharmaceutical companies. The CRITIC method is a method used to calculate the objective weights of the criteria considered in multi-criteria decision making problems. The objective weight obtained by this method synthesizes the contrast intensity of each criterion and the contradiction between the criteria. The contrast intensity of the criterion is taken as the standard deviation and the correlation coefficient is used to calculate the discrepancy between the criteria. In the CRITIC method, it is aimed to extract the information found in the evaluation criteria by analyzing the decision matrix analytically. The algorithm of the CRITIC Method is as shown below^24^:

- *Step 1* Normalizing the Decision Matrix. Rʹ = (rʹij)m × n matrix (i: alternative, j: criterion) is done with normalized The Eq. (1).
  1 $$ r_{ij}^{^{\prime}} = \left\{ \begin{gathered} \frac{{r_{ij} - r_{j}^{ - } }}{{r_{j}^{ + } - r_{j}^{ - } }},\,\,for\, benefit\,the\, criteria, \hfill \\ \frac{{r_{j}^{ + } - r_{ij} }}{{r_{j}^{ + } - r_{j}^{ - } }},\,\,for\, cost\,the\, criteria\,, \hfill \\ \end{gathered} \right.\,{\text{Here}}\,\begin{array}{*{20}l} {r_{j}^{ - } = \mathop {\min }\limits_{i} \,r_{ij} \,\,ve\,\,r_{j}^{ + } = \mathop {\max }\limits_{i} r_{ij} } \\ {{\kern 1pt} (i = 1,2,....,m;j = 1,2,....,k)} \\ \end{array} . $$
- *Step 2* Calculation of the standard deviations ($\sigma_{j}$) of the criteria: Calculation of the standard deviation is done by The Eq. (2). Here, $\overline{r}_{j}$, j. is the column mean of the criterion.
  2 $$ \sigma_{j} \, = \sqrt {\frac{{\sum\limits_{i = 1}^{m} {(r_{ij}^{{\prime}} - \overline{r}_{j} )^{2} } }}{m}} \;{\text{here}}\;\overline{r}_{j} = \frac{{\sum_{i = 1}^{m} {r_{ij}^{{\prime}} } }}{m}. $$
- *Step 3* Determining the correlation ($\rho_{jk}$) between criteria. The correlation value between the j criterion and the k criterion is calculated by The Eq. (3).
  3 $$ \rho_{jk} = \frac{{\sum_{i = 1}^{m} {(r_{ij}^{{\prime}} - \overline{r}_{j} )(r_{ik}^{^{\prime}} - \overline{r}_{k} )} }}{{\sqrt {\sum_{i = 1}^{m} {(r_{ij}^{^{\prime}} - \overline{r}_{j} )^{2} \sum_{i = 1}^{m} {(r_{ik}^{{\prime}} - \overline{r}_{k} )^{2} } } } }},\;(k = 1,2,....,k;j = 1,2,....,k). $$
- *Step 4* Calculation of the amount of information for each criterion. The amount of information is calculated by The Eq. (4).
  4 $$ c_{j} = \sigma_{j} \sum\limits_{k = 1}^{n} {(1 - \rho_{jk} )} ,\;(k = 1,2,....,k;j = 1,2,....,k). $$
- *Step 5* Determination of criterion weights. The criterion weights are determined using The Eq. (5).
  5 $$ w_{j} = \frac{{c_{j} }}{{\sum_{j = 1}^{n} {c_{j} } }},\;(j = 1,2,....,k). $$

#### ORESTE method

ORESTE was developed as a new MCDM method by Roubens in 1982 as an alternative to the ELECTRE method^25^. ORESTE is basically a simple ranking method in which the superiority of the alternatives over each alternative is determined according to each criterion. That is, this method decides in favor of the superior alternative, regardless of the relative amount of difference between the alternatives^25^. The method has opposite processing steps unlike the ELECTRE method. In the first stage (ORESTE I), a full preliminary row of alternatives is made. In the second stage (ORESTE II), since some values of these front rows are incomparable and indifferent values, a conflicted analysis is performed and some parts are removed^7^. This method is more suitable for problems involving qualitative data as it only considers the ranking of alternatives and criteria. It can also be used for problems involving quantitative or mixed data^14^. In this study, since the second phase of the ORESTE method is handled with a different approach, it is also introduced in the “Proposed method” section. The first phase of the ORESTE method consists of three steps and is as follows:

- *Step 1* Ranking of Alternatives and Criteria. A weak rank of criteria is made in which indifference (I) and preference (P) relations are taken into account. This rank is a preference structure with a weak or complete front rank in which the importance relationship of the criterion is determined. Likewise, the weak rank of the alternatives on the basis of each criterion is determined in accordance with the definition of mean rank proposed by Besson. Then, using the mean rank, a new value is assigned to each alternative of this weak rank to form a global (real-exact) rank.
- *Step 2* Calculation of projection distances. An initial distance point is created in the position table according to the values calculated by the distances for each alternative. These distances are also used to compare alternatives by considering the importance relationship of the criterion. In Eqs. (6) and (7), it allows to determine the relative positions of the alternatives according to a random origin point based on the rank value of the alternative and the superiority of the criteria. Here; Dj(a) a alternative j. projection distance value for the criterion, r(Cj) j. besson ordinal value of the criterion and rCj(i) is j. is the besson ordinal value of the i.alternative considering the criterion. Pasijn and Leysen^7^ used the nonlinear projection formula in The Eq. (8) to determine projection distances.
  6 $$ {\text{If a P}}_{{\text{j}}} {\text{b}}, \;{\text{D}}_{{\text{j}}} \left( {\text{a}} \right) \, < {\text{ D}}_{{\text{j}}} \left( {\text{b}} \right) \quad \left( {{\text{j}} = {1},{2}, \ldots {\text{k}}} \right), $$
  7 $$ {\text{If}}\;{\text{rC}}_{{1}} \left( {\text{a}} \right)\; = \;{\text{rC}}_{{2}} \left( {\text{b}} \right) \;{\text{and}}\; {\text{C}}_{{1}} {\text{P C}}_{{2}} , \;{\text{D}}_{{1}} \left( {\text{a}} \right) \, < {\text{ D}}_{{2}} \left( {\text{b}} \right), $$
  8 $$ {\text{D}}_{{{\text{ji}}}} \left( {\text{a}} \right) \, = \, \left[ {{\acute{\alpha}}{\text{ r}}\left( {{\text{C}}_{{\text{j}}} } \right)^{{\text{R}}} + \, \left( {{1} - \, {\acute{\alpha}}} \right){\text{ rC}}_{{\text{j}}} \left( {\text{a}} \right)^{{\text{R}}} } \right]^{{{1}/{\text{R}}}} \quad \left( {{\text{i}} = {1},{2}, \ldots {\text{m}}} \right). $$

In The Eq. (8), ά is the weighted factor. If ά = 0.5, the sequence of alternatives and criteria is equally important. R is a parameter chosen by the decision makers. Below are the different R values and their meanings:

- R = 1: average rank (arithmetic mean);
- R =  − 1: rank harmonic mean;
- R = 2: quadratic average rank;
- R =  − ∞: min(r(Cj), rCj(a))R =  + ∞: max(r(Cj), rCj(a)).

The larger the R value, the more weight is given to the larger r(Cj) and rCj(a) terms. After measuring the distance distance for each criterion and alternative, the alternative and criterion clustering can be done by designing a position matrix on the fundamental diagonal. Thus, those in better positions are positioned to the left, while those in weaker positions are positioned to the right.

- *Step 3* Ranking of all projections (Global Rank): Since the distances previously determined for each alternative are not clear values, it may be misleading to make the actual ranking according to these values. Therefore, a new rank value is assigned to all values in the position matrix and the global rank is created.

### Proposed method

To solve the conflict problem in the second step of the ORESTE method, it is proposed to create the preference density matrix of the alternatives by using the CRITIC weights of the criteria, and to calculate the net flow values with reference to the PROMETHEE II method (C-ORESTE). C-ORESTE is a method developed to eliminate the incompatibility problem in the second stage of the ORESTE method, taking into account the CRITIC weight method. For the two alternatives a and b to be discussed in the ORESTE Method, if a is slightly better than b for some criteria and bad for the other criterion, a is no different from b. In this case, it will be impossible to conclude that one alternative is better than the other in terms of preference relationship, and these two alternatives can be considered as equivalent to each other. However, if alternative a is much better than alternative b for some criteria and very bad for other criteria, a conflicting situation arises this time. In other words, if alternative b is good for some criteria and bad for some criteria, an incomparable situation arises. This raises a problem of incompatibility for two incomparably different alternatives. In order to solve this problem, it has been suggested that in the second step of the ORESTE method, the preference density matrix of the alternatives should be created using the CRITIC weights of the criteria, and in the third step, net flow values should be calculated based on the PROMETHEE II method according to the preference density matrix. In this way, since the Global weak rank values are taken into account in determining the preference density among the alternatives, it is aimed to obtain more effective results by taking into account the degree of contradiction between the criteria, and to obtain a new preference structure according to the net flow values and to rank the alternatives completely. The C-ORESTE method consists of 2 stages, C-ORESTE I and C-ORESTE II.

#### C-ORESTE II method

At this stage, conflict analysis is done by taking into account the CRITIC weights. The difference of C-ORESTE II from the ORESTE II method is that it normalizes according to CRITIC weights instead of mean weights. The main purpose of the conflict analysis is to test the suitability of evaluating the alternatives with the ORESTE method. Because the global rank of the alternatives obtained in the simple ranking step does not include some cases. Differences or incomparability between different alternatives are analyzed in more detail. The two alternatives are indistinguishable when both alternatives are (almost) good or (almost) bad for the same criteria. When both alternatives are good or bad for different criteria, these two alternatives are not comparable. In other words, if the first alternative is very good for these criteria, the second alternative is very bad, or vice versa, these two alternatives are incomparable^8^. C-ORESTE II method consists of two steps and is as follows:

- *Step 1* Establishing the normalized preference density. For the conflicted analysis, it is proposed to first calculate the normalized preference density with The Eq. (9). The difference of this equation from the normalized equation proposed by Pasijn and Leysen^7^ is that the CRITIC weights of the criteria are taken into account. Here, Cʹ(a,b) denotes the preference density of alternative a over alternative b, rj(a) denotes the global rank of alternative a in j. criterion, and wj denotes CRITIC weight of j. criterion.
  9 $$ C{\kern 1pt} (a,b) = \frac{{\sum\limits_{{j:\,\,a\,p_{j} \,b}} {w_{j} (r_{j} (b) - r_{j} (a))} }}{(m - 1)k}. $$
- Prof. It corresponds to 1/k in the normalized preference density equation proposed by Wj, Pasijn and Leysen^7^ in The Eq. (10). In the normalized equality proposed by Pasijn and Leysen^7^, each criterion has equal weight.
  10 $$ \sum\limits_{j}^{k} {w_{j} } = \frac{1}{k} + \frac{1}{k} + \cdots + \frac{1}{k} = 1\quad \left( {\frac{1}{k},{\text{ as the amount of criteria}}} \right). $$
- *Step 2* Performing the conflicted analysis. For the conflicted analysis, Pastijn and Leysen^7^ needed to calculate 3 thresholds to distinguish between the alternatives indifference (I), incomparable (R), and preference (P)^7^:
  - *Threshold β* → A threshold based on the minimum value that can be certain to be superior to another alternative. Threshold *β*; gives the relationship between indifference (I) and preference (P) and is calculated The Eq. (11) as:
    11 $$ \beta \le \, \, \frac{1}{(m - 1)k}. $$
  - Threshold C* → The minimum condition at which one alternative can be equal to another alternative, a threshold that gives the relationship between indifference (I) and inomparable (R). The values in the mean rank index of the alternatives are taken into account and action is taken accordingly. In the mean rank array, the 1 rank superiority of alternative a over alternative b corresponds to the number of criteria (k) value in the global array. According to Pastijn and Leysen^7^; when analyzing this equal threshold, alternative a has to be superior to alternative b by half the number of criteria, since there is a minimum situation that can occur between two alternatives. Likewise, alternative b is superior to alternative a by half the number of criteria. In this case; The density of preference for alternative a to alternative b becomes [(k/2) × k]/[(n − 1)k2]. Therefore, the value to be used when analyzing the equality between the alternatives is calculated The Eq. (12) as:
    12 $$ C* \le \, {\kern 1pt} \frac{1}{2(m - 1)}. $$
  - *Threshold γ* → A threshold that gives the relationship between the degree of closeness of a value to another value and the preference (P) and incomparable(R). This threshold should be less than the net preference density of alternative y a relative to alternative b and is calculated The Eqs. (13) and (14) as:
- In the dual criteria decision matrix;
  13 $$ \gamma < \, {\kern 1pt} \tfrac{{C(a,b){\kern 1pt} - {\kern 1pt} C(b,a){\kern 1pt} }}{C(b,a)}\, {\kern 1pt} = \, {\kern 1pt} \frac{((k + 2)/2) \times k - ((k - 2)/2) \times k}{{((k - 2)/2) \times k}}\, {\kern 1pt} = \, {\kern 1pt} \frac{4}{k - 2}. $$
- In a single criterion decision matrix;
  14 $$ \gamma < \, {\kern 1pt} \tfrac{{C(a,b){\kern 1pt} - {\kern 1pt} C(b,a){\kern 1pt} }}{C(b,a)}\, {\kern 1pt} = \, {\kern 1pt} \frac{((k + 1)/2) \times k - ((k - 1)/2) \times k}{{((k - 1)/2) \times k}}\, {\kern 1pt} = \, {\kern 1pt} \frac{2}{k - 1}. $$

As a result of these threshold tests, there are 4 situations between any two alternatives. Therefore, the ORESTE flowchart^7^ is applied for these threshold tests and the conflict test between the two alternatives.

#### C-ORESTE III method

When the incompatibility situation occurs between the alternatives as a result of the conflicted analysis, a complete ranking can be made by considering the normalized preference density matrix. For this, net flow values are obtained by taking the difference of positive and negative superiorities, as in the PROMETHEE II method^26^, and a complete ranking is made according to these values. The reason why the PROMETHEE II method is taken as reference is that the normalization equation in The Eq. (15) corresponds to the type 3 preference function in the PROMETHEE method. The Eq. (15) are as follows:

15 $$ C{\kern 1pt} (a,b) = \, \left\{ {\begin{array}{*{20}c} {\frac{{\sum\limits_{{j:\,\,a\,p_{j} \,b}} {w_{j} (r_{j} (b) - r_{j} (a))} }}{(m - 1)k}\, ,} & {\sum\limits_{{j:\,\,a\,p_{j} \,b}} {w_{j} (r_{j} (b) - r_{j} (a))} \, {\kern 1pt} < \, {\kern 1pt} (m - 1)k} \\ {1,} & {\sum\limits_{{j:\,\,a\,p_{j} \,b}} {w_{j} (r_{j} (b) - r_{j} (a))} \, \, \ge \, {\kern 1pt} (m - 1)k} \\ \end{array} } \right\}. $$

**Figure 1:**
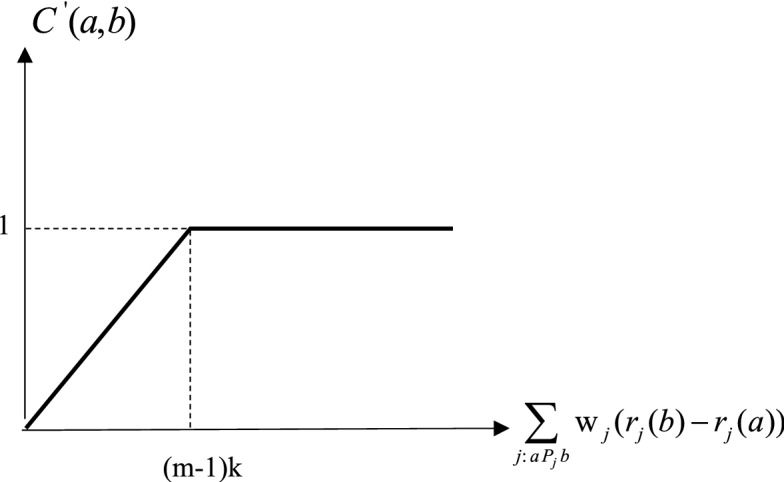
ORESTE II normalized preference density function (PROMETHEE Type 3 Preference Function).

ORESTE II normalized preference density function function corresponding to type 3 preference function of PROMETHEE method is as in Fig. 1. Figure 1 are as follows.

C-ORESTE III allows for complete ranking of alternatives. For the full ranking of the alternatives, the net flow values are determined by The Eq. (16). Here, C(*a*) is the net flow value of alternative a, $\sum C(a,x)$ the sum of preference density (positive superiority) in row values of alternative a, and $\sum C(x,a)$ in column values of alternative a. refers to the sum of preference density (negative superiority).

16 $$ C(a) = \, \sum {C(a,x)\, - {\kern 1pt} } \sum {C(x,a)\, } . $$

With the Eq. (17) given for a and b alternatives, alternative a is preference to alternative b, while The Eq. (18) and alternatives a and b are indifferent.

17 $$ \begin{array}{*{20}c} {\text{a P b}} & {C\left( a \right) \, > \, C\left( b \right),} \\ \end{array} $$

18 $$ \begin{array}{*{20}c} {{\text{a }} = {\text{ b}}} & {C\left( a \right) \, = \, C\left( b \right)} \\ \end{array} . $$

**Figure 2:**
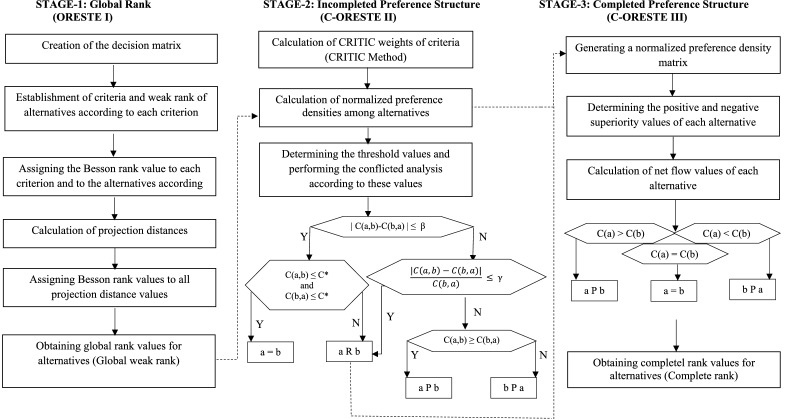
The flow chart of the C-ORESTE method.

The flow chart of the C-ORESTE method, which includes global ranking (ORESTE I), conflicted analysis (C-ORESTE II), and complete ranking (C-ORESTE III), is as in Fig. 2. Figure 2 shows the three-phase flowchart of the C-ORESTE method and explained as follows: In the first stage, the global order of the alternatives is obtained with the ORESTE I method. These rank values are input to the C-ORESTE II method in the second step, and the normalized preference density is calculated among the alternatives, taking into account the CRITIC weights of the criteria. According to these normalized preference densities and threshold values, a preference structure is created among the alternatives. That is, an alternative is either superior, equal, or incomparable. If there is an incomparable situation, the normalized preference intensities obtained from the C-ORESTE II method are input to the C-ORESTE III method and the complete order is obtained by obtaining the net flow values according to these values. In summary, ORESTE-1; basic row, C-ORESTE II; CRITIC weighted incomplete preference structure and C-ORESTE III; Provides a complete preference structure with reference to PROMETHEE II.

### Application

In order to test the validity and applicability of the C-ORESTE method, an application was made to the logistics center location problem within the scope of green logistics. Green logistics is a term that emerged with the aim of minimizing the environmental problems that arise as a result of logistics activities^4^. This definition brings the concept of green logistics to the fore in practice in order to reduce environmental problems in the logistics sector and to ensure sustainability. When the studies on green logistics are examined, it has been determined that it is mostly related to the fields of supply chain, storage, transportation, etc.

In this study, optimum location selection was made considering the suitability of 9 alternative logistics centers in Turkey (Balıkesir (A1), Erzurum (A2), Kahramanmaraş (A3), Samsun (A4), Istanbul (A5), Eskişehir (A6), Denizli (A7), Kocaeli (A8), Uşak (A9)) for green logistics activities. The data required for the application were obtained by conducting fieldwork with the help of an expert team. However, the criteria taken into account in the selection of green logistics location were determined by the expert team consisting of industry and academic staff within the framework of the concept of sustainability and environmentalism. These criteria and their definitions are given below:

- Capacity (Tons): It is the load capacity of the logistics center in tons. (C1)
- Total Area (m^2^): It is the total area of the logistics center. (C2)
- Sun (kwh\m^2^-year): It is the total amount of solar radiation. (C3)
- Wind Speed (km/h): It is the annual wind speed of the region where the logistics center is located. (C4)
- Nearest port (km): It is the distance of the closest port to the logistics center. (C5)
- Nearest airline (km): It is the distance of the nearest airline to the logistics center. (C6)
- Annual precipitation (mm): It is the annual precipitation received by the region where the logistics center is located. (C7)
- Annual sunshine duration (hours): It is the annual sunshine duration of the region where the logistics center is located. (C8)
- Pm10: The amount of particulate matter pollutant in the air of the city where the logistics center is located (Pm10-2018 data). PM10 refers to dust particles smaller than 10 µm in diameter. (C9)
- Carbon footprint (Tons): It is the amount of damage caused by human activities to the environment in terms of the amount of greenhouse gas produced, measured in units of carbon dioxide. (C10)

**Figure 3:**
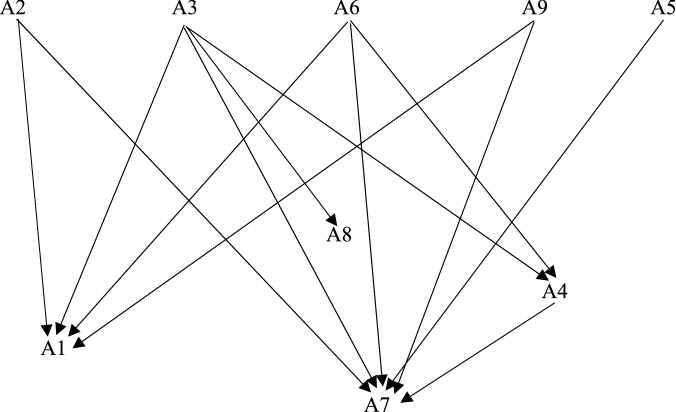
C-ORESTE II structure.

In order to evaluate the green logistics center, the decision matrix consisting of 9 alternative logistics centers and 10 criteria to be considered in the application of optimal location selection with the C-ORESTE method is given in Table 2. The application steps for this section are as follows:

1. Finding the criterion weights with the CRITIC method: The criterion weights were determined by applying the processing steps of the CRITIC method to the data in the decision matrix. Microsoft EXCEL software was used for this. Weight values for each criterion are given in Table 3. According to these values, CRITIC is the carbon footprint (C10) criterion with the highest weight. This criterion has the highest contradiction density and provides the most information in calculating the preference density between alternatives.
2. Obtaining the weak global rankings of the alternatives with the ORESTE I method: In the first step of this step, the Besson weak rank of the criteria was made by the decision maker and then the Besson weak rank of the alternatives was made on the basis of each criterion according to the data in the decision matrix. The Besson weak rank matrix is given in Table 4. In the second step, the projection distances of the Besson weak rank values were calculated using The Eq. (7). For this, R = 2 and á = 0.5 are taken. In the third step, a global rank matrix of alternatives to these projection distance values was created. The global rank matrix is given in Table 5.
3. Performing the conflicted analysis with the C-ORESTE-II method: The preference density matrix and the conflicted matrix were obtained by applying the C-ORESTE II processing steps to the global rank matrix. For the conflicted analysis, þ = 0.0125, C* = 0.0625, y = 0.5 were calculated. The preference density matrix is given in Table 6 and the conflicted matrix is given in Table 7. Accordingly, A2, A3, A6, A9 and A5 alternatives, which no alternative is superior, are incomparable among themselves. A3 is the alternative with the most superiority and A7 is the alternative with the most superiority. The preference structure created as a result of the conflicted test is as in Fig. 3. The reason for this is that the Global rank values based on criteria are close to each other and there is a conflict. This creates an incomplete preference structure.
4. Obtaining complete rank values for C-ORESTE-III method: By applying C-ORESTE III processing steps to the preference density matrix, positive superiority, negative superiority and net flow values of each alternative were obtained. These values are given in Table 8 and the complete ranking of the alternatives is made according to the net flow values. Accordingly, A3 has the highest net flow value and Kahramanmaraş is the most suitable province for green logistics activities. Then, respectively, A2 (Erzurum) and A9 (Uşak) alternatives. In the last rank is the A7-Denizli alternative. Thus, the preference structure that could not be completed in C-ORESTE II was completed with C-ORESTE III. The fact that Kahramanmaraş comes to the forefront is that it has the top ranking in the global ranking values and contains fewer contradictions than other alternatives. However, since the CRITIC weights of the criteria are close to each other, it has been determined that there is not much effect on the rank values of the alternatives. Since the CRITIC method makes objective weighting according to the contradiction of the values in the decision matrix, its use in the ORESTE II method gave more effective and reliable results.

**Table 2:** Decision matrix.

Criteria →	C1	C2	C3	C4	C5	C6	C7	C8	C9	C10
Alternative↓	Benefit	Benefit	Benefit	Benefit	Cost	Cost	Cost	Benefit	Cost	Cost
A1	1.000.000	211.000	2	6.5	110	8.13	605	80.6	46	15.8
A2	437.000	350.000	5	6,5	171	10.8	43.4	82.6	41	14.94
A3	1.900.000	805.000	5	5	126	4.11	719.7	80.8	92	15.73
A4	1.156.000	258.000	1	5.5	20	10	718.3	62.4	48	14.63
A5	2.000.000	220.000	1	7	10	27	391.9	81.5	44	15.8
A6	1.400.000	541.000	3	5.5	237	10	374.2	72.2	44.5	14.71
A7	500.000	125.300	3	3	163	23	589	87.6	68	14.77
A8	2.000.000	694.000	1	5	15	12	816	67.6	40	15.8
A9	246.000	140.000	4	6	6.35	202.95	557	87.2	46	14.77

**Table 3:** CRITIC weights of criteria.

Criteria →	C1	C2	C3	C4	C5	C6	C7	C8	C9	C10
Weight	0.099	0.088	0.111	0.089	0.108	0.089	0.081	0.103	0.078	0.153

**Table 4:** Besson weak rank matrix.

	C1	C2	C3	C4	C5	C6	C7	C8	C9	C10
Weak rank	7.5	6	2	4	9	10	7.5	5	3	1
A1	6	7	6	2.5	5	2	4	6	5.5	8
A2	8	4	1.5	2.5	8	5	9	3	2	5
A3	3	1	1.5	7.5	6	1	2	5	9	6
A4	5	5	8	5.5	4	3.5	3	9	7	1
A5	1.5	6	8	1	2	8	7	4	3	8
A6	4	3	4.5	5.5	9	3.5	8	7	4	2
A7	7	9	4.5	9	7	7	5	1	8	3.5
A8	1.5	2	8	7.5	3	6	1	8	1	8
A9	9	8	3	4	1	9	6	2	5.5	3.5

**Table 5:** Global rank matrix.

	C1	C2	C3	C4	C5	C6	C7	C8	C9	C10
A1	64.5	59.5	26.5	12.5	73.5	70.0	51.5	39.5	24.5	42.0
A2	79.5	33.0	3.5	12.5	86.0	81.0	84.5	21.0	6.5	17.5
A3	44.5	22.5	3.5	51.5	77.5	69.0	38.0	32.0	62.5	22.5
A4	56.5	39.5	47.0	30.5	66.5	75.5	44.5	73.5	35.0	1.0
A5	36.5	49.0	47.0	10.0	59.5	89.0	71.5	28.0	11.0	42.0
A6	51.5	29.0	14.5	30.5	88.0	75.5	79.5	55.0	16.0	2.0
A7	71.5	77.5	14.5	66.5	82.0	87.0	56.5	17.5	54.0	8.5
A8	36.5	26.5	47.0	51.5	62.5	83.0	34.0	61.0	5.0	42.0
A9	84.5	68.0	6.5	20.0	58.0	90.0	64.5	19.0	24.5	8.5

**Table 6:** Preference density matrix.

	A1	A2	A3	A4	A5	A6	A7	A8	A9
A1	0.000	0.081	0.086	0.109	0.070	0.094	0.153	0.114	0.078
A2	0.149	0.000	0.122	0.183	0.147	0.091	0.178	0.204	0.085
A3	0.159	0.127	0.000	0.161	0.183	0.124	0.171	0.155	0.154
A4	0.127	0.133	0.106	0.000	0.131	0.066	0.180	0.110	0.117
A5	0.096	0.105	0.136	0.139	0.000	0.128	0.210	0.093	0.106
A6	0.151	0.080	0.108	0.105	0.159	0.000	0.181	0.161	0.121
A7	0.109	0.065	0.054	0.117	0.140	0.080	0.000	0.165	0.030
A8	0.123	0.145	0.090	0.101	0.075	0.113	0.218	0.000	0.163
A9	0.139	0.078	0.142	0.160	0.141	0.125	0.135	0.215	0.000

**Table 7:** Conflict matrix.

	A1	A2	A3	A4	A5	A6	A7	A8	A9
A1	I	<	<	R	R	<	R	R	<
A2	>	I	R	R	R	R	>	R	R
A3	>	R	I	>	R	R	>	>	R
A4	R	R	<	I	R	<	>	R	R
A5	R	R	R	R	I	R	>	R	R
A6	>	R	R	>	R	I	>	R	R
A7	R	<	<	<	<	<	I	R	<
A8	R	R	<	R	R	R	R	I	R
A9	>	R	R	R	R	R	>	R	I

**Table 8:** Positive superiority, negative superiority and net flow values (complete ranking).

	$\sum C(a,x)$	$\sum C(a,x)$	*C(a)*	Rank
A1	0.785	1.054	− 0.269	8
A2	1.159	0.815	0.345	2
A3	1.235	0.844	0.391	1
A4	0.970	1.075	− 0.104	6
A5	1.013	1.047	− 0.033	5
A6	1.066	0.821	0.245	4
A7	0.759	1.425	− 0.666	9
A8	1.028	1.218	− 0.189	7
A9	1.135	0.854	0.281	3

Since PROMETHEE II is referenced in the proposed method, the results obtained from C-ORESTE and PROMETHEE II methods were compared. The aim is to test the validity of the C-ORESTE III method. In the ORESTE method, the preference density matrix is obtained by normalizing the global rank matrix. This process corresponds to the 3rd type preference function of the PROMETHEE II method. Therefore, PROMETHEE II processing steps^26^ were applied to the global rank values in Table 5 and the results were compared. Here, type 3 preference function and CRITIC weights are considered for all criteria. Comparative results of both methods are given in Table 9. According to these results, the order of alternatives was the same.

**Table 9:** C-ORESTE and PROMETHEE II comparative analysis.

Alternative →		A1	A2	A3	A4	A5	A6	A7	A8	A9
C-ORESTE III	*C(a)*	− 0.269	0.345	0.391	− 0.104	− 0.033	0.245	− 0.666	− 0.189	0.281
	Rank	8	2	1	6	5	4	9	7	3
PROMETHEE II	Q_net_	− 0.0332	0.0417	0.0492	− 0.0118	− 0.003	0.0306	− 0.0859	− 0.0208	0.0333
	Rank	8	2	1	6	5	4	9	7	3

## Conclusions

In this study, a new integrated method including CRITIC and ORESTE methods from MCDM methods and aiming to solve the problem of inconsistency in the ORESTE method was been proposed. To solve the inconsistency problem in the ORESTE method, the preference density matrix of the alternatives was created by using the CRITIC weights. Then, by summing the row and column values in the preference density matrix, positive–negative superiority values and the difference between these values were calculated, and negative superiority values were calculated. The PROMETHEE II method is taken as a reference for these processes. The reason for taking the PROMETHEE II method as a reference is that the normalized preference density equation proposed by Pastijn and Leysen^7^ corresponds to the PROMETHEE type-3 preference function. In addition, the results obtained as a result of the application were compared with the results of the PROMETHEE II method. According to these results, it was remarkable that the alternative ranks were the same, and it was determined that the C-ORESTE III method was effective and valid.

The proposed method aims to add a new integrated method to the literature by completing the preference structure that could not be completed in the second phase of the ORESTE method with the addition of another step. The results obtained as a result of the application revealed that the method is valid and can be easily applied to other decision-making problems. However, the proposed method has some limitations. Ranking the criteria in the first stage and weighting them in the second stage can create a disadvantage for the decision-maker. Because the criteria are evaluated both subjectively and objectively. This situation can create problems in the application of problems involving linguistic data. In the future, the following studies can be carried out regarding the proposed method:

- Other weighting methods can be used instead of the CRITIC method.
- For fuzzy decision making problems, the proposed method in fuzzy framework can be developed.

## Data Availability

The datasets used and/or analysed during the current study available from the corresponding author on reasonable request.

## References

[1] Kutlu BS, Abalı YA, Eren T (2012). Çok Ölçütlü Karar Verme Yöntemleri ile Seçmeli Ders Seçimi. Sosyal Bilimler.

[2] Çınar Y, Kabak M (2020). Çok Kriterli Karar Vermenin Esasları. Çok Kriterli Karar Verme Yöntemleri MS Excel ve Software Çözümlü Uygulamalar.

[3] Yerlikaya MA, Arıkan F (2016). Constructing the performance effectiveness order of SME supports programmes via Promethee and Oreste techniques. J. Faculty Eng. Architect. Gazi Univ..

[4] Sbihi A, Eglese RW (2010). Combinatorial optimization and Green Logistics. Ann. Oper. Res..

[5] Roubens M (1980). Analyse et agrégation des préférences: Modélisation, ajustement et résumé de données relationnelles. Rev. Beige de Recherche Oper. de Stat. d’Inf..

[6] Roubens M (1982). Preference relations on actions and criteria in multicriteria decision making. Eur. J. Oper. Res..

[7] Pastijn H, Leysen J (1989). Constructing an outranking relation with ORESTE. Math. Comput. Model..

[8] Pastjin H, Leener I (2002). Selecting land mine detection strategies by means of outranking MCDM techniques. Eur. J. Oper. Res..

[9] Givescu O (2007). The Oreste’s method in the multicriteria’s decision process for the management of tourism field. Manag. Econ. Ser. Manag..

[10] Matějček M, Brožová H (2011). Multiple Attributes Analysis of Vegetable Production. MCBANTA'11. Nouras Barbu Lupulescu, Snejana Yordanova, and Valeri Mladenov.

[11] Chatterjee P, Chakraborty S (2013). Advanced manufacturing systems selection using ORESTE method. Int. J. Adv. Oper. Manag..

[12] Jafari H, Noshadi E, Khosheghbal B (2013). Ranking ports based on competitive ındicators by using ORESTE method. Int. Res. J. Appl. Basic Sci..

[13] Eroğlu E, Yıldırım BF, Özdemir M (2014). Çok Kriterli Karar Vermede ORESTE Yöntemi ve Personel Seçiminde Uygulanması. Yönetim Dergisi.

[14] Tuş Işık A (2016). QUALIFLEX and ORESTE methods for the ınsurance company selection problem. J. Oper. Res. Stat. Econometr. Manag. Inf. Syst..

[15] Luo S, Liang W, Zhao G (2020). Likelihood-based hybrid ORESTE method for evaluating the thermal comfort in underground mines. Appl. Soft Comput..

[16] Wang X, Gou X, Xu Z (2020). Assessment of traffic congestion with ORESTE method under double hierarchy hesitant fuzzy linguistic environment. Appl. Soft Comput..

[17] Zheng Q, Liu X, Wang W (2021). An extended ınterval type-2 fuzzy ORESTE method for risk analysis in FMEA. Int. J. Fuzzy Syst..

[18] Long Y, Liao H (2021). A social participatory allocation network method with partial relations of alternatives and it sapplication in sustainable food supply chain selection. Appl. Soft Comput..

[19] Akman G, Pamuk KC, Karabıçak Ç (2021). Yeni Ürün Geliştirme Sürecinde Bulanık AHP & ORESTE Bütünleşik Yöntemi ile Tedarikçi Seçimi: Savunma Sanayisinde Bir Uygulama. Bilecik Şeyh Edebali Üniv. Fen Bilimleri Dergisi.

[20] Shi K, Liu Y, Liang W (2022). An extended ORESTE approach for evaluating rockburst risk under uncertain environments. Mathematics.

[21] Zhang H, Gao H, Liu P (2022). Assessment of regional economic restorability under the stress of COVID-19 using the new interval type-2 fuzzy ORESTE method. Complex Intell. Syst..

[22] Qin J, Liang Y, Martinez L (2022). ORESTE-SORT: A novel multiple criteria sorting method for sorting port group competitiveness. Ann. Oper. Res..

[23] Diakoulaki D, Mavrotas G, Papayannakis L (1995). Determining objective weights in multiple criteria problems: The CRITIC method. Comput. Oper. Res..

[24] Yerlikaya MA (2021). Belirsiz Sipariş Toplama Sistemlerinde Ürün Atama Kriterlerinin Pisagor Bulanık CRITIC Yöntemiyle Önceliklendirilmesi. Bulanık Çok Kriterli Karar Verme Yöntemleri MS Excel ve Software Çözümlü Uygulamalar.

[25] Doumpos M, Figueira JR (2019). A multicriteria outranking approach for modeling corporate credit ratings: An application of the electre Tri-nC method. Omega.

[26] Brans JP, Vincke P (1985). A preference ranking organization method: The PROMETHEE method for MCDM. Manag. Sci..

