# Publisher Correction: Solution proposal for completed preference structure in ORESTE method

**DOI:** 10.1038/s41598-023-33766-z

**Published:** 2023-04-21

**Authors:** Mehmet Akif Yerlikaya, Kürşat Yildiz, Büşra Nur Keskin

**Affiliations:** 1 Deparment of Industrial Engineering, Bitlis Eren University, Bitlis, Turkey; 2 Deparment of Civil Engineering, Gazi University, Ankara, Turkey; 3 Deparment of Traffic Planning and Application, Gazi University, Ankara, Turkey

Correction to: *Scientific Reports* 10.1038/s41598-023-31561-4, published online 23 March 2023

The original version of this Article contained an error in Table 9, where values for A1, A4, A5, A7 and A8 were incorrect in row C-ORESTE III: C(*a*). The correct and incorrect values appear below.

Incorrect:

**Table:** 

Alternative →		A1	A4	A5	A7	A8
C-ORESTE III	C(*a*)	0.269	0.104	0.033	0.666	0.189

Correct:

**Table:** 

Alternative →		A1	A4	A5	A7	A8
C-ORESTE III	C(*a*)	− 0.269	− 0.104	− 0.033	− 0.666	− 0.189

The original Article has been corrected.

